# Supplementary material for: A Critical View of the Application of the APEX Software (Aqueous Photochemistry of Environmentally-Occurring Xenobiotics) to Predict Photoreaction Kinetics in Surface Freshwaters
Source: Molecules. 2019 Dec 18;25(1):9. doi: 10.3390/molecules25010009 (PMC7017383; doi:10.3390/molecules25010009)
Supplement: Supplementary file 1 [file molecules-25-00009-s001.zip › 3.2.4_gcc-4.4.0.zip!/3.2.4_gcc-4.4.0/3.2.4_gcc-4.4.0/doc/octave/html/interpreter/Arithmetic.html]

Arithmetic - Untitled


Next: Linear Algebra,
Previous: Matrix Manipulation,
Up: Top


---

## 17 Arithmetic

Unless otherwise noted, all of the functions described in this chapter
will work for real and complex scalar, vector, or matrix arguments. Functions
described as mapping functions apply the given operation individually to
each element when given a matrix argument. For example,

```
     sin ([1, 2; 3, 4])
          =>  0.84147   0.90930
              0.14112  -0.75680
```

- Exponents and Logarithms- Complex Arithmetic- Trigonometry- Sums and Products- Utility Functions- Special Functions- Coordinate Transformations- Mathematical Constants
